# Non-randomized comparison between revascularization and deferral for intermediate coronary stenosis with abnormal fractional flow reserve and preserved coronary flow reserve

**DOI:** 10.1038/s41598-021-88732-4

**Published:** 2021-04-28

**Authors:** Doosup Shin, Joo Myung Lee, Seung Hun Lee, Doyeon Hwang, Ki Hong Choi, Hyun Kuk Kim, Joon-Hyung Doh, Chang-Wook Nam, Eun-Seok Shin, Masahiro Hoshino, Tadashi Murai, Taishi Yonetsu, Hernán Mejía-Rentería, Tsunekazu Kakuta, Javier Escaned, Bon-Kwon Koo

**Affiliations:** 1 Division of Cardiovascular Medicine, Department of Internal Medicine, University of Iowa Carver College of Medicine, Iowa City, IA USA; 2 Division of Cardiology, Department of Internal Medicine, Heart Vascular Stroke Institute, Samsung Medical Center, Sungkyunkwan University School of Medicine, 81 Irwon-ro, Gangnam-gu, Seoul, 06351 Korea; 3 Division of Cardiology, Department of Internal Medicine, Chonnam National University Hospital, Gwangju, Korea; 4 Department of Internal Medicine and Cardiovascular Center, Seoul National University Hospital, 101 Daehang-ro, Chongno-gu, Seoul, 110-744 Korea; 5 Department of Internal Medicine and Cardiovascular Center, Chosun University Hospital, University of Chosun College of Medicine, Gwangju, Korea; 6 Department of Medicine, Inje University Ilsan Paik Hospital, Goyang, Korea; 7 Department of Medicine, Keimyung University Dongsan Medical Center, Daegu, Korea; 8 Division of Cardiology, Ulsan Medical Center, Ulsan, Korea; 9 Division of Cardiovascular Medicine, Tsuchiura Kyodo General Hospital, Ibaraki, Japan; 10 Department of Cardiovascular Medicine, Tokyo Medical and Dental University, Tokyo, Japan; 11 Cardiovascular Institute, Hospital Clinico San Carlos, Madrid, Spain; 12 Centro Nacional de Investigaciónes Cardiovasculares Carlos III (CNIC), Madrid, Spain

**Keywords:** Cardiology, Interventional cardiology

## Abstract

Limited data are available regarding comparative prognosis after percutaneous coronary intervention (PCI) versus deferral of revascularization in patients with intermediate stenosis with abnormal fractional flow reserve (FFR) but preserved coronary flow reserve (CFR). From the International Collaboration of Comprehensive Physiologic Assessment Registry (NCT03690713), a total of 330 patients (338 vessels) who had coronary stenosis with FFR ≤ 0.80 but CFR > 2.0 were selected for the current analysis. Patient-level clinical outcome was assessed by major adverse cardiac events (MACE) at 5 years, a composite of all-cause death, target-vessel myocardial infarction (MI), or target-vessel revascularization. Among the study population, 231 patients (233 vessels) underwent PCI and 99 patients (105 vessels) were deferred. During 5 years of follow-up, cumulative incidence of MACE was 13.0% (31 patients) without significant difference between PCI and deferred groups (12.7% vs. 14.0%, adjusted HR 1.301, 95% CI 0.611–2.769, *P* = 0.495). Multiple sensitivity analyses by propensity score matching and inverse probability weighting also showed no significant difference in patient-level MACE and vessel-specific MI or revascularization. In this hypothesis-generating study, there was no significant difference in clinical outcomes between PCI and deferred groups among patients with intermediate stenosis with FFR ≤ 0.80 but CFR > 2.0. Further study is needed to confirm this finding.

Clinical Trial Registration: International Collaboration of Comprehensive Physiologic Assessment Registry (NCT03690713; registration date: 10/01/2018).

## Introduction

Coronary physiologic assessment using pressure-derived fractional flow reserve (FFR) has become a standard method for identifying functionally significant epicardial coronary artery stenosis^1–3^. Based on multiple clinical trials^4–6^, FFR-guided percutaneous coronary intervention (PCI) for intermediate stenosis has been incorporated into current guidelines and clinical practice^1–3^. In the FAME II trial, however, 73.0% of medically managed patients with intermediate stenosis and abnormal FFR ≤ 0.80 did not experience any adverse outcomes during 5-year follow-up^6^. Furthermore, impairment of myocardial perfusion or ischemia is not only determined by the extent of epicardial stenosis, but also by the alteration of coronary microvasculature which cannot be fully assessed by FFR^7,8^. Coronary flow reserve (CFR), defined as maximal coronary blood flow divided by control flow at rest, is a physiologic index reflecting myocardial reserve vasodilator capacity^9^. Previous studies indicated that depressed CFR was strongly associated with functionally significant disease that is prone to cause ischemia^10^, and preserved CFR was shown to have excellent negative predictive value for excluding high risk coronary artery disease^11^. Since discordance between FFR and CFR is not uncommon in patients with intermediate stenosis and both indices can provide complementary information regarding underlying pathophysiology, the combined measurement of coronary flow and pressure has been suggested for a better understanding of the disease and appropriate therapeutic decision making^12–15^. Furthermore, recent DEFINE-FLOW study showed comparable clinical outcome between deferred patients with FFR ≤ 0.80 and CFR ≥ 2.0 and revascularized patients with FFR ≤ 0.80 and CFR < 2.0^16^.

Considering that the recent ISCHEMIA trial did not show benefit of invasive strategy compared with initial conservative strategy among patients with stable coronary disease and moderate or severe ischemia^17^, a clinically relevant question would be whether preserved CFR in patients with intermediate stenosis and abnormal FFR could potentially affect patient’s prognosis according to different treatment strategies. In this regard, the comparative efficacy between PCI and deferral for lesion with FFR ≤ 0.80 and CFR ≥ 2.0 needs more clarification, however, limited data are available regarding comparative prognosis between PCI and deferral strategies in this patient population. Therefore, we sought to investigate clinical outcomes after PCI or deferral of revascularization among patients with intermediate coronary stenosis with FFR ≤ 0.80 and CFR > 2.0.

## Methods

### Study design and population

The study population was derived from the International Collaboration of Comprehensive Physiologic Assessment Registry (NCT03690713; registration date 10/01/2018), a pooled cohort of 3 prospective registries which have been previously published^18–20^. Participants of each registry were prospectively enrolled from tertiary medical centers in Korea (Seoul National University Hospital, Samsung Medical Center, Inje University Ilsan Paik Hospital, Keimyung University Dongsan Medical Center, and Ulsan University Hospital), Japan (Tsuchiura Kyodo General Hospital), and Spain (Hospital Clinico San Carlos), respectively. All patients underwent clinically-indicated invasive coronary angiography and comprehensive physiologic assessments for at least one vessel with intermediate stenosis (40% to 80% of diameter stenosis)^18–20^. The same exclusion criteria were applied to all 3 registries, and patients with hemodynamic instability or left ventricular dysfunction (ejection fraction < 30%) and culprit vessels of acute coronary syndrome were excluded. Individual patient data were collected using standardized spreadsheets. For all variables included, standardized definitions were used.

**Figure 1:**
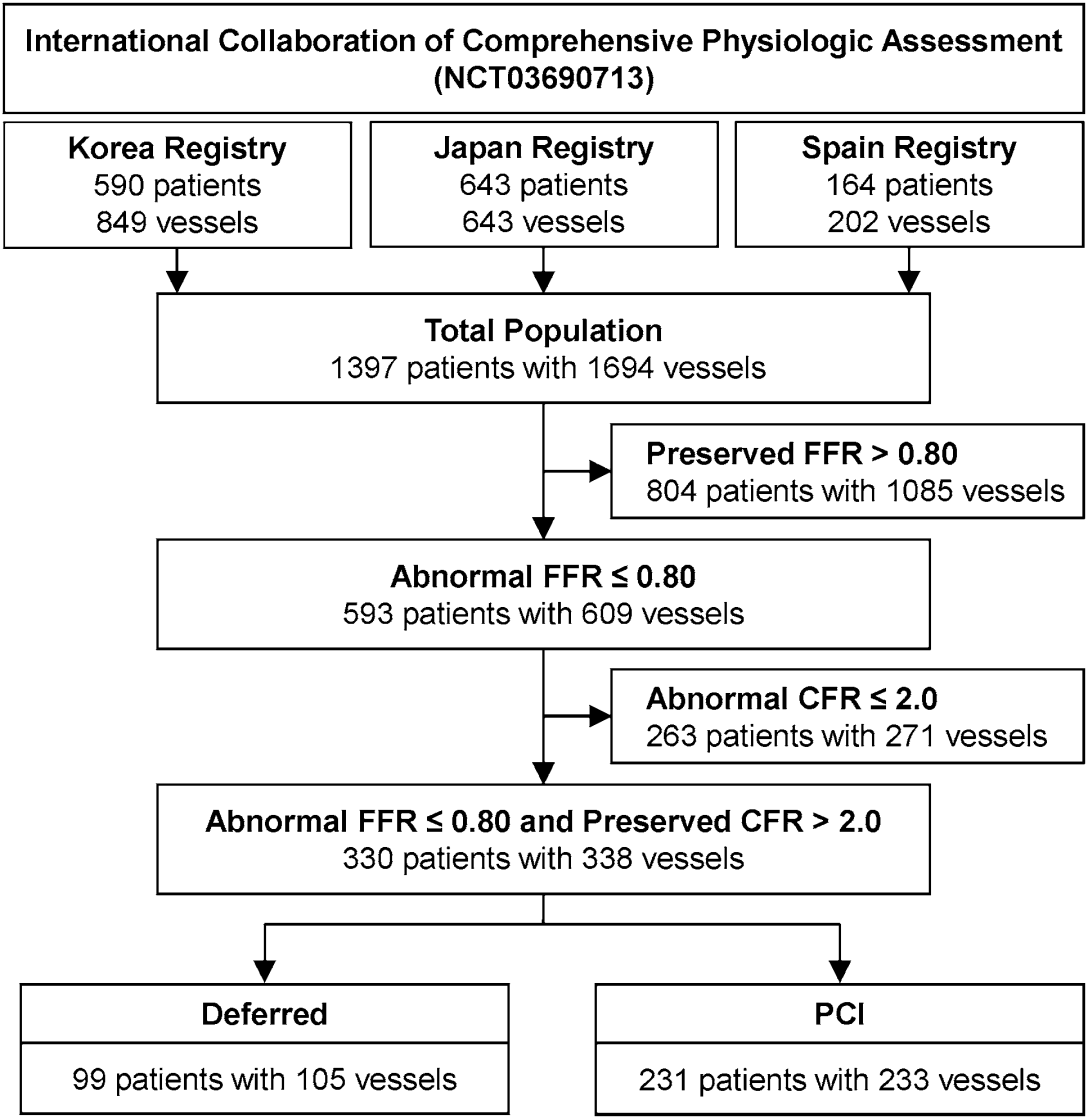
Study flow. From the international cohort of 3 prospective registries, 330 patients (338 vessels) with abnormal FFR ≤ 0.80 but preserved CFR > 2.0 were included in the current study. *CFR* Coronary flow reserve, *FFR* Fractional flow reserve, *PCI* Percutaneous coronary intervention.

Among a total of 1,397 patients (1,694 vessels) from the initial pooled cohort, 330 patients (338 vessels) who had intermediate stenosis with abnormal FFR ≤ 0.80 but preserved CFR > 2.0 were included in the current study (Fig. 1). Study protocols were designed in accord with the Declaration of Helsinki (2013). All patients gave informed consent and the study protocol was authorized by Seoul National University Hospital Institutional Review Board.

### Coronary angiography and physiologic measurements

All coronary angiograms were performed using standard techniques and analyzed at local core laboratories in a blinded fashion^18–20^. Percent diameter stenosis, minimum luminal diameter, reference-vessel size, and lesion length were measured^21^. All coronary physiologic indices were measured after diagnostic angiograms. After zeroing and equalizing to the aortic pressure, a pressure–temperature sensor guide wire (Abbott Vascular, St. Paul, MN, USA) was positioned at the distal segment of the target vessel to measure the physiologic indices^21^. Intracoronary nitrate (100 or 200 µg) was administered before each physiologic measurement. To derive resting mean transit time (Tmn), a thermodilution curve was obtained using 3 injections of room-temperature saline (4 mL each)^19,21^. Hyperemia was induced by intravenous infusion of adenosine (140 µg/kg/min) through a peripheral or central vein^19,21^. Hyperemic proximal aortic pressure (Pa), distal arterial pressure (Pd), and hyperemic Tmn were measured during sustained hyperemia after the pressure curve reached a nadir point^21,22^. The hyperemic period was recognized by a decreased Pd/Pa pattern and a left shift in the Tmn^21^. FFR was calculated as hyperemic Pd/Pa, at the lowest average of 3 consecutive beats during maximal hyperemia^21^. CFR was calculated as the resting Tmn divided by the hyperemic Tmn^13^. After measurements were completed, the guide wire was pulled back to the guide catheter, and the presence of a pressure drift was checked^19,21^. Invasive physiologic indices were cross-checked and confirmed by principal investigators of each registry.

For lesions with abnormal FFR (≤ 0.80), PCI was recommended according to the current guidelines^21^. However, the final decision for PCI was at the discretion of the operator. Current analysis used only pre-PCI physiologic indices^21^.

### Data collection, follow up, and clinical outcomes

Patient demographics, cardiovascular risk factors, and clinical diagnoses were recorded at the time of index procedure. Clinical data were obtained using standardized spreadsheets at outpatient clinic visits or by telephone contact if needed. Median follow-up duration of the study population was 1286 days (interquartile range: 733–1693 days). The primary outcome was patient-level MACE during 5 years of follow-up, a composite of all-cause death, target-vessel MI, or target-vessel revascularization. Vessel-level clinical outcome was also assessed by vessel-specific MI or vessel-specific revascularization. All clinical outcomes were defined according to the Academic Research Consortium report. All deaths were considered cardiac unless an undisputable non-cardiac cause was present. Periprocedural MI was not accounted as a clinical event. Target vessel MI was defined as spontaneous MI which occurred in the initially interrogated vessel. Revascularization was additionally adjudicated as to whether the event occurred in the initially interrogated vessel.

### Statistical analysis

Analyses were performed on a per-patient basis for clinical characteristics and primary outcome (and its individual components) and on a per-vessel basis for vessel-related parameters and vessel-level clinical outcomes. If a patient underwent multivessel assessments, the vessel with the lowest FFR value was selected as a representative vessel of that patient for per-patient analysis.

Categorical variables were presented as number with relative frequency (percentage) and continuous variables as mean with standard deviation or medians with first and third quartiles (Q1-Q3) according to their distributions determined by Kolmogorov–Smirnov test. The cumulative incidence of clinical events was presented as Kaplan–Meier estimate and compared using a log-rank test. Univariable and multivariable Cox proportional hazard regressions were used to calculate unadjusted and adjusted HR and 95% CI. Adjusted variables included age, gender, diabetes mellitus, hypertension, hypercholesterolemia, current smoking, and presentation with acute coronary syndrome. The assumption of proportionality was assessed graphically by log-minus-log plot, and Cox proportional hazard models for all clinical outcomes satisfied the proportional hazards assumption.

To further adjust for uneven distribution of baseline characteristics between the PCI and deferred groups, multiple sensitivity analyses were performed. First, PS-adjusted Cox proportional hazard regression analyses were performed. PS was calculated from multiple logistic regression models after adjusting for age, sex, hypertension, diabetes, hypercholesterolemia, smoking, presentation with acute coronary syndrome, target vessel location, pre-PCI %DS, lesion length, FFR and CFR. Second, the analyses were repeated in a PS-matched cohort. Third, IPW Cox proportional hazard regression models were used.

To identify independent predictors of MACE and a composite of vessel-specific MI or revascularization, multivariable Cox proportional hazard regression model was constructed using all clinically relevant variables and those with a p-value of < 0.05 from the univariate analyses. In addition, comparison of clinical outcomes between the PCI and deferred groups was performed within subgroups, according to age (< 70 and ≥ 70 years), sex (female and male), diabetes, hypercholesterolemia, current smoking, %DS (> 70% and ≤ 70%), pre-PCI FFR (> 0.75 and ≤ 0.75), and pre-PCI CFR (≥ 4.0 and < 4.0). The interaction between treatment effect and these covariates was assessed using a Cox proportional hazard regression. All probability values were 2-sided, and p-values < 0.05 were considered statistically significant.

## Results

### Characteristics of the study population

**Figure 2:**
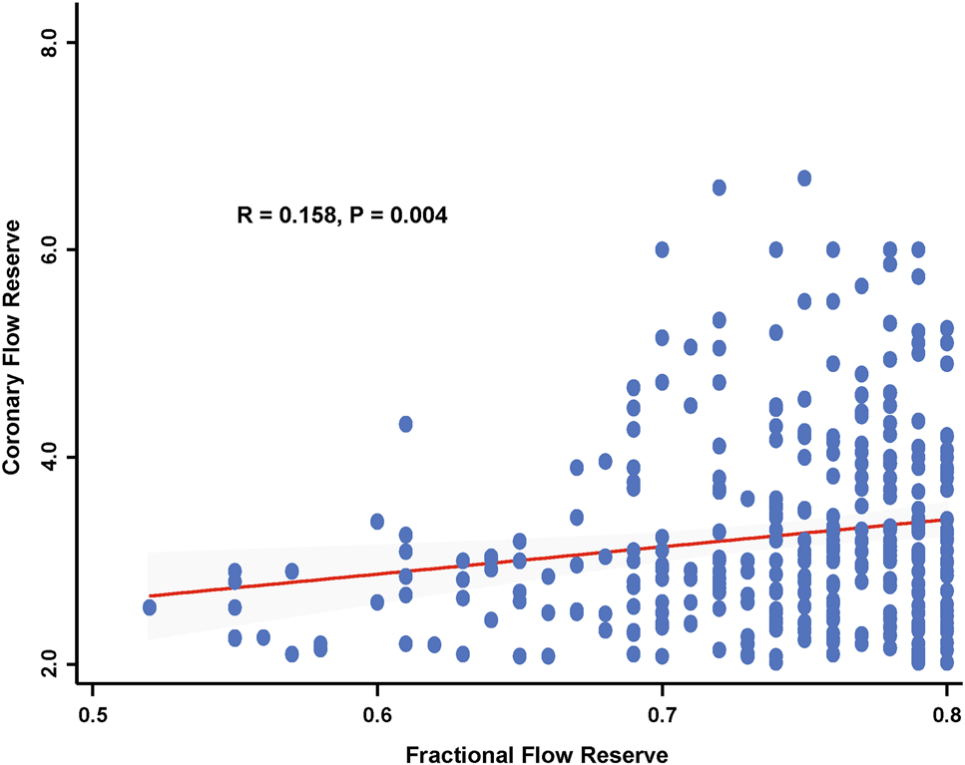
Association between FFR and CFR. The association between FFR and CFR among patients with FFR ≤ 0.80 and CFR > 2.0 is shown. *CFR* Coronary flow reserve, *FFR* Fractional flow reserve.

Baseline characteristics of the study population are shown in Table 1. The mean age was 64.3 ± 9.7 years and 11.2% were female. Mean percent diameter stenosis (%DS) was 53.8 ± 13.6, and pre-PCI FFR and CFR were 0.75 (Q1-Q3: 0.71–0.78) and 3.0 (Q1-Q3: 2.5–3.8), respectively. Distributions of FFR, CFR, and %DS are presented in Supplementary Fig. 1. There was modest correlation between pre-PCI FFR and pre-PCI CFR (*r* = 0.158, *P* = 0.004) (Fig. 2).

**Table 1:** Baseline characteristics of patients and lesions.

	Total	PCI	Deferred	*P* value
Per-patient analysis (n = 330)	330	231	99	
**Demographics**				
Age	64.3 ± 9.7	65.1 ± 9.7	62.3 ± 9.3	0.016
Female	37 (11.2)	25 (10.8)	12 (12.1)	0.879
**Cardiovascular risk factors**				
Hypertension	220 (66.7)	151 (65.4)	69 (69.7)	0.524
Diabetes mellitus	109 (33.0)	81 (35.1)	28 (28.3)	0.283
Hyperlipidemia	225 (68.2)	157 (68.0)	68 (68.7)	1.000
Current smoker	74 (22.4)	51 (22.1)	23 (23.2)	0.931
Family history of CAD	30 (9.9)	21 (10.1)	9 (9.5)	1.000
**Clinical presentation**				0.297
Acute coronary syndrome	39 (11.8)	24 (10.4)	15 (15.2)	
Stable ischemic heart disease	291 (88.2)	207 (89.6)	84 (84.9)	
Per-vessel analysis (n = 338)	338	233	105	
**Target vessel location**				0.014
Left anterior descending artery	274 (81.3)	182 (78.1)	92 (88.5)	
Left circumflex artery	20 (5.9)	13 (5.6)	7 (6.7)	
Right coronary artery	43 (12.8)	38 (16.3)	5 (4.8)	
**Quantitative coronary angiography**				
Reference vessel diameter, mm	2.77 (2.38–3.15)	2.75 (2.37–3.13)	2.88 (2.38–3.19)	0.597
Minimal luminal diameter, mm	1.26 (1.01–1.56)	1.17 (0.94–1.43)	1.48 (1.17–1.71)	< 0.001
Diameter stenosis, %	53.8 ± 13.6	56.7 ± 13.0	47.7 ± 12.8	< 0.001
Lesion length, mm	12.56 (8.00–19.68)	13.24 (8.55–20.35)	10.00 (6.16–18.06)	0.026
**Invasive physiologic indices**				
FFR, Pre-PCI	0.75 (0.71–0.78)	0.74 (0.69–0.77)	0.78 (0.75–0.79)	< 0.001
CFR, Pre-PCI	3.00 (2.50–3.80)	2.96 (2.50–3.54)	3.14 (2.58–3.90)	0.065

Values are mean ± standard deviations, median (interquartile range), or number (%).

*CAD* Coronary artery disease, *CFR* Coronary flow reserve, *FFR* Fractional flow reserve, *PCI* Percutaneous coronary intervention.

Among the study population, PCI was performed in 231 patients (70.0%) and was deferred in 99 patients (30.0%). There was no significant difference in cardiovascular risk factors between PCI and deferred groups (Table 1). Compared with deferred vessels, the revascularized vessels showed higher %DS (56.7 ± 13.0 vs. 47.7 ± 12.8, *P* < 0.001), longer lesion length (13.24 [Q1-Q3: 8.55–20.35] vs. 10.00 [Q1-Q3: 6.16–18.06], *P* = 0.026), and lower pre-PCI FFR (0.74 [Q1-Q3: 0.69–0.77] vs. 0.78 [Q1-Q3: 0.75–0.79], *P* < 0.001). Proportions of the vessels with pre-PCI FFR of 0.76–0.80, 0.71–0.75, and ≤ 0.70 were 48.8%, 28.1%, and 23.1%, respectively (Deferred group: 72.4%, 21.0%, and 6.7%, respectively; PCI group: 38.2%, 31.3%, and 30.5%, respectively).

Among patients in whom revascularization was deferred, PCI was deferred based on operator’s discretion and specific reasons are presented in Table 2. In the deferred group, there was no significant difference in demographics, cardiovascular risk factors, coronary angiographic findings, and invasive physiologic indices between the patients with and without major adverse cardiac events (MACE; Supplementary Table 1).

**Table 2:** Specific reasons for deferral of revascularization in patients with FFR ≤ 0.80 and CFR > 2.0.

Reasons for deferral of revascularization	Proportion in deferral group
Minimal stenosis on angiography	32.3% (32)
Good exercise performance with tolerable symptom and negative non-invasive tests	17.2% (17)
Diffuse disease without focal stenosis in FFR pullback curve	15.2% (15)
Gray zone FFR with preserved CFR	15.2% (15)
Not suitable for PCI based on clinical condition or technical reason	9.1% (9)
No angiographic progression since previous angiography	8.1% (8)
Small myocardial territory or limited viability	3.0% (3)

Values are proportion (patient number).

*CFR* Coronary flow reserve, *FFR* Fractional flow reserve, *PCI* Percutaneous coronary intervention.

### Clinical outcomes between PCI and deferred groups

**Figure 3:**
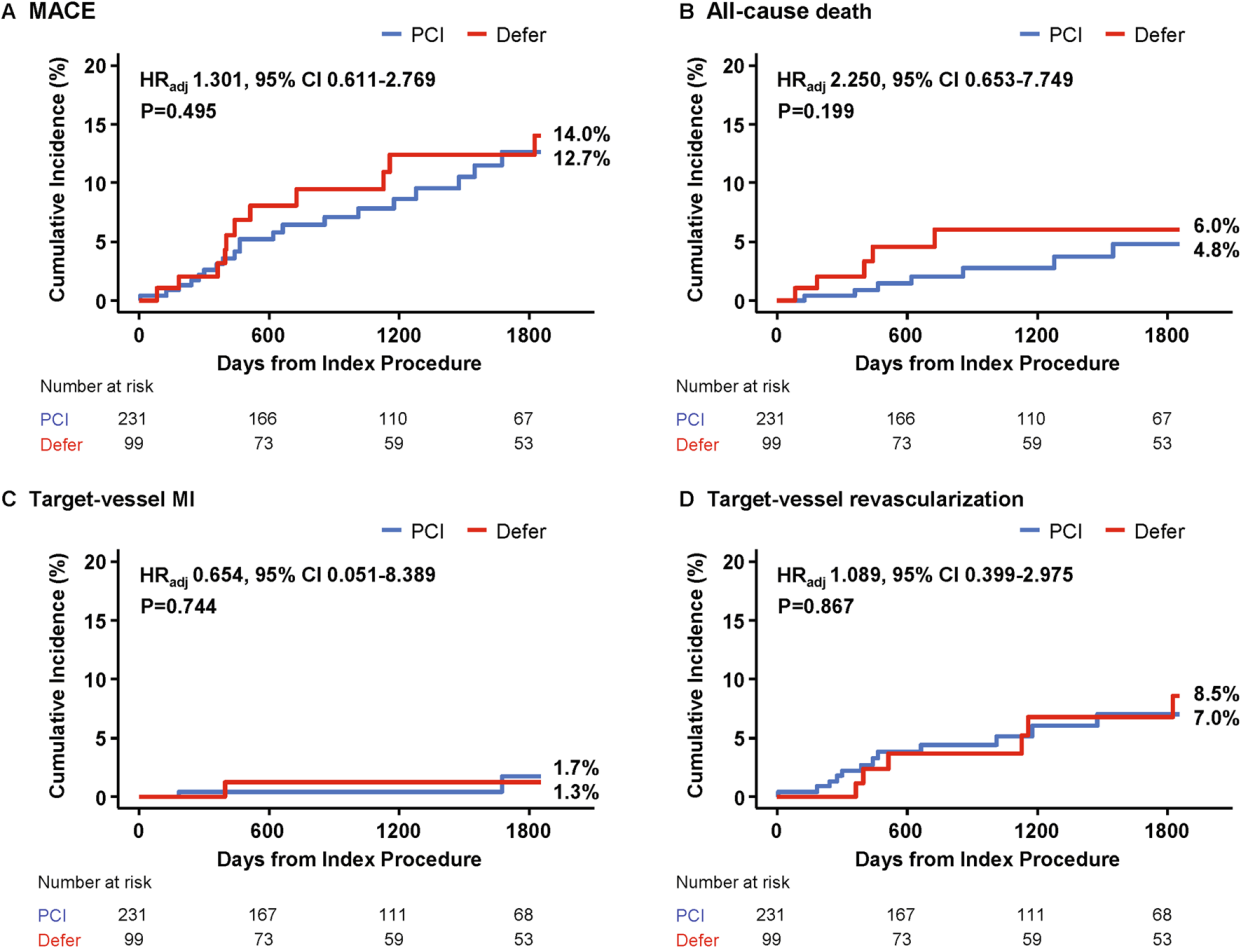
Comparison of major adverse cardiac events according to treatment strategy. Kaplan–Meier curves and cumulative incidence of MACE (and its individual components) were compared according to the treatment strategies (PCI or deferral of revascularization). Adjusted HR and 95% CI were calculated based on multivariable Cox proportional hazard regression model. Adjusted variables included age, sex, diabetes mellitus, hypertension, hypercholesterolemia, current smoking, and presentation with acute coronary syndrome. *CI* Confidence interval, *HR*_*adj*_ Adjusted hazard ratio, *MACE* Major adverse cardiac event, *MI* Myocardial infarction, *PCI* Percutaneous coronary intervention.

During 5 years of follow-up, cumulative incidence of patient-level MACE among the total population was 13.0% (31 patients). There was no significant difference in MACE between PCI and deferred groups (12.7% vs. 14.0%, adjusted hazard ratio [HR] 1.301, 95% confidence interval [CI] 0.611–2.769, *P* = 0.495). Furthermore, the risks of individual components of MACE were similar between the 2 groups, including all-cause death, target-vessel myocardial infarction (MI), and target-vessel revascularization (Table 3 and Fig. 3). Multiple sensitivity analyses using propensity score (PS) adjustment, inverse probability weighted (IPW) adjustment, and PS matched analysis also showed no significant difference in the risk of MACE or its individual components between the 2 groups (Table 4).

**Table 3:** Comparison of clinical outcomes according to treatment strategy.

Per-patient analysis	PCI (N = 231)	Deferred (N = 99)	Unadjusted HR (95% CI)	*P* value	Adjusted HR* (95% CI)	*P* value
MACE^†^	12.7 (20)	14.0 (11)	1.156 (0.553–2.419)	0.700	1.301 (0.611–2.769)	0.495
All-cause death	4.8 (7)	6.0 (5)	1.561 (0.494–4.928)	0.448	2.250 (0.653–7.749)	0.199
Target-vessel MI	1.7 (2)	1.3 (1)	0.985 (0.088–11.03)	0.990	0.654 (0.051–8.389)	0.744
Target-vessel revascularization	7.0 (12)	8.5 (6)	1.056 (0.395–2.823)	0.913	1.089 (0.399–2.975)	0.867

Data expressed as cumulative incidence of clinical outcomes and number of events. Cumulative incidence of clinical outcomes represents Kaplan–Meier estimates during median follow-up of 1286.0 days (Q1-Q3 733.0–1693.0 days). *P* values for log-rank test in survival analysis.

*Adjusted for age, sex, diabetes mellitus, hypertension, hypercholesterolemia, current smoking, and acute coronary syndrome.

^†^MACE included all-cause death, target-vessel MI, and target-vessel revascularization.

*CI* Confidence interval, *HR* Hazard ratio, *MACE* Major adverse cardiac events, *MI* Myocardial infarction, *PCI* Percutaneous coronary intervention.

**Table 4:** Sensitivity analysis regarding clinical outcomes according to treatment strategy.

Per-patient analysis	PS*-adjusted HR (95% CI)	*P* value	IPW adjusted HR (95% CI)	*P* value	PS*-matched HR (95% CI)	*P* value
MACE^†^	1.754 (0.717–4.288)	0.218	1.011 (0.448–2.284)	0.979	0.995 (0.402–2.461)	0.990
All-cause death	2.277 (0.523–9.920)	0.273	1.364 (0.380–4.895)	0.634	1.942 (0.354–10.64)	0.445
Target-vessel MI	0.445 (0.032–6.190)	0.546	1.505 (0.094–23.98)	0.772	0.799 (0.048–13.22)	0.876
Target-vessel revascularization	1.893 (0.606–5.914)	0.273	0.958 (0.325–2.831)	0.939	0.908 (0.291–2.833)	0.868

*Propensity score was calculated based on multiple logistic regression model after adjusting for age, sex, hypertension, diabetes, hypercholesterolemia, smoking, clinical presentation, vessel location, percent diameter stenosis, lesion length, pre-intervention fractional flow reserve, and pre-intervention coronary flow reserve.

^†^MACE included all-cause death, target-vessel MI, and target-vessel revascularization.

*CI* Confidence interval, *HR* Hazard ratio, *IPW* Inverse probability weight, *MI* Myocardial infarction, *MACE* Major adverse cardiac events, *PS* Propensity score.

Similar results were observed in per-vessel analysis. There was no significant difference in vessel-level clinical outcomes between PCI and deferred groups with regard to a composite of vessel-specific MI or revascularization (8.1% vs. 8.0%, adjusted HR 0.932, 95% CI 0.321–2.712, *P* = 0.898) (Supplementary Table 2 and Supplementary Fig. 2). Sensitivity analyses using PS adjustment, IPW adjustment, and PS matching also showed consistent results (Supplementary Table 3).

### Subgroup analysis and independent predictors of composite outcomes

**Figure 4:**
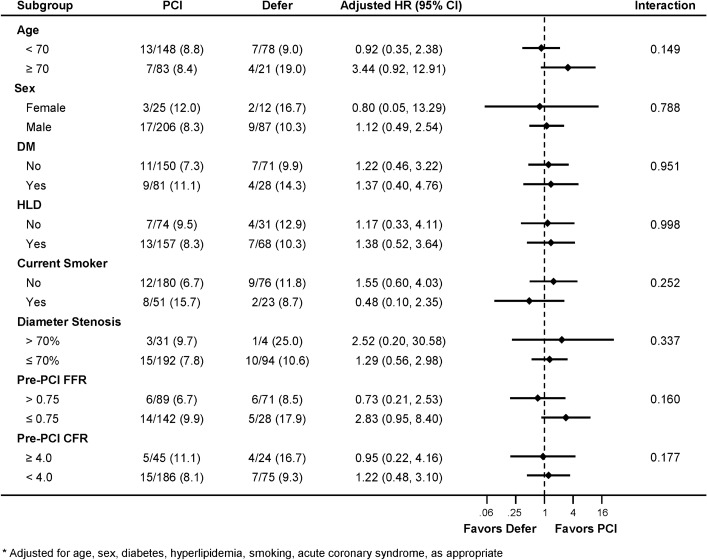
Subgroup analysis for major adverse cardiac events. Comparison of major adverse cardiac event according to treatment strategy (PCI or deferral of revascularization) was performed within various subgroups. Adjusted HR and 95% CI were calculated based on multivariable Cox proportional hazard regression model. Adjusted variables included age, sex, diabetes, hypercholesterolemia, current smoking, and presentation with acute coronary syndrome, as appropriate. *CFR* Coronary flow reserve, *CI* Confidence interval, *DM* Diabetes mellitus, *FFR* Fractional flow reserve, *HLD* Hyperlipidemia (hypercholesterolemia), *HR* Hazard ratio, *PCI* Percutaneous coronary intervention.

The prognostic impact of revascularization deferral on clinical outcomes among the various subgroups was investigated. There was no significant difference in MACE between PCI and deferred groups across all subgroups without significant interaction (Fig. 4). Similarly, there was no difference in vessel-specific MI or revascularization between the 2 groups across all subgroups without significant interaction (Supplementary Fig. 3).

In multivariable Cox proportional hazard models, treatment strategy (PCI or deferral) was not independently associated with either MACE (adjusted HR 0.463, 95% CI 0.187–1.144, *P* = 0.095) or a composite of vessel-specific MI or revascularization (HR 0.807, 95% CI 0.161–4.045, *P* = 0.795) (Supplementary Tables 4 and 5).

## Discussion

In the current study, we investigated clinical outcomes after PCI or deferral of revascularization among patients with intermediate coronary stenosis and abnormal FFR (≤ 0.80) but preserved CFR (> 2.0). The main findings were as follows. First, there was no significant difference in MACE and its individual components between PCI and deferred groups. These results were consistently observed in multiple sensitivity analyses. Second, per-vessel analysis also showed no significant difference in vessel-specific MI or revascularization between the 2 groups. Third, similar risk of MACE or vessel-specific MI or revascularization was observed in various subgroups. Furthermore, treatment strategy (PCI or deferral) was not independently associated with either MACE or vessel-specific MI or revascularization.

FFR has been used as a standard method to identify functionally significant epicardial coronary stenosis with the potential to induce myocardial ischemia^1–3^, and FFR-guided PCI strategy has been validated by multiple landmark clinical trials^4–6^. In FAME 2 trial, PCI plus medical therapy was superior to medical therapy alone among patients with stable coronary artery disease and functionally significant stenoses determined by FFR ≤ 0.80^6^. In this study, however, it should be noted that 73.0% of medically managed patients with functionally significant intermediate stenosis had not experienced any adverse outcomes during 5-year follow-up without PCI^6^.

This raised an important question regarding how to improve contemporary FFR-guided PCI strategy, which requires a thorough understanding of the physiologic basis. In this regard, it is important to recognize that FFR is a pressure-derived surrogate of relative flow reserve^23^, but not a direct measurement of coronary flow impairment which is a major determinant of myocardial ischemia^12^. Also, myocardial contractile function depends on coronary flow but not on coronary perfusion pressure^12,14,24^. In fact, coronary flow can be low despite normal coronary pressure, and vice versa^14,25^. Therefore, comprehensive interpretation of both pressure- and flow-based indexes would be important for a full physiological evaluation of the target vessel territories^25^.

CFR is a well-validated, flow-based physiologic index which permits a comprehensive assessment of the coronary circulation^12,14^. Disagreement between flow-based CFR and pressure-based FFR has been described in up to 40% of lesions^12–15,26^, which reflects underlying physiology including atherosclerotic disease pattern, individual susceptibility of flow regulation, and microvascular function. Furthermore, studies have demonstrated the impact of a disagreement between FFR and CFR on prognosis. Among patients with deferred lesions with preserved FFR, low CFR was associated with a significantly higher risk of adverse outcomes compared with preserved CFR^13,15,19,27^. Conversely, patients with deferred lesions with low FFR but preserved CFR showed similar clinical outcomes compared to those with concordantly preserved values^19,27^. These results support that coronary flow, which can be represented by CFR, would be a complementary tool to FFR-guided strategy, not only to enhance understanding of the disease but also to guide treatment decision-making. However, relatively large impact of hemodynamic status on CFR and high measurement variability has been major hurdles in using CFR in daily practice^28^.

Within this context, a clinically relevant question pertains to what the optimal treatment strategy for intermediate stenoses with discordant FFR and CFR values would be. However, little is known regarding the prognosis after initial PCI or deferral of revascularization among patients with intermediate stenosis with abnormal FFR but preserved CFR. The current study demonstrated that there was no significant difference in clinical outcomes according to the initial treatment strategies among those patients. This result is in line with prior studies which showed similar prognosis of deferred lesions with FFR ≤ 0.80 but preserved CFR compared to those with concordantly preserved values^19,27^. Recently, DEFINE-FLOW study (NCT02328820) evaluated comparative prognosis of patients according to FFR and CFR. In this study, only patients with FFR ≤ 0.80 and CFR ≤ 2.0 were revascularized, conversely, patients with FFR ≤ 0.80 and CFR > 2.0 were deferred. At 2 years from index procedure, MACE rates were comparable between deferred patients with FFR ≤ 0.80 and CFR > 2.0 and revascularized patients with FFR ≤ 0.80 and CFR ≤ 2.0 (10.8% vs. 14.4%, respectively)^16^. These findings suggest possible role of comprehensive physiologic assessment. From a hemodynamics perspective, a large pressure drop across an epicardial stenosis could be due to an unimpaired coronary vasodilator response resulting in a large trans-stenotic flow-induced pressure gradient^14^. In such cases, initial revascularization of the epicardial stenosis may not always provide significant benefits in increasing coronary flow in subtended myocardial territory. However, it should be noted that patients with FFR < 0.75 showed non-significant trend favoring PCI over medical treatment. Considering the continuous relationship of FFR with the potential risk of clinical events^29,30^, the prognostic impact of PCI according to CFR values in patients with FFR ≤ 0.80 might be different according to the ranges of FFR value. In DEFINE-FLOW, all patients with FFR ≤ 0.80 and CFR > 2.0 were managed medically and no one underwent PCI according to study protocol. Therefore, the current study, where initial deferral strategy was compared with PCI among the patients with FFR ≤ 0.80 and CFR > 2.0, was different from DEFINE-FLOW and added unique values to the clinically important questions. Both studies are hypothesis-generating and further randomized trials will be required due to inherent limitations.

When interpreting the results of the current study, it is important to acknowledge reasons for deferral of revascularization determined by individual operators. Common reasons included minimal stenosis on angiography (32.3%), good exercise performance with tolerable symptoms and negative non-invasive tests (17.2%), and diffuse disease without focal stenosis in FFR pullback curve (15.2%). Also, 15.2% of the deferred cases were due to gray zone FFR (0.75–0.80) with preserved CFR, and 76.8% of patients in the deferred group showed gray zone FFR. Prior study from IRIS-FFR registry reported that there was no significant difference in clinical outcomes between initial revascularization and deferral strategies for coronary stenosis with gray zone FFR^31^. In their study, higher risk of periprocedural MI after PCI was offset by higher risk of target vessel revascularization with deferral among the lesions with gray zone FFR^31^. In the current study, 56.1% of the total population and 76.8% of the patients in the deferred group had gray zone FFR. In contrast to the study from IRIS-FFR registry, however, the risk of target vessel revascularization was not significantly higher in the deferred group than the PCI group. These results suggest an additional value of CFR in the risk stratification of patients with gray zone FFR in whom the decision for revascularization could be individualized without an increased risk of target vessel revascularization after deferral.

With the recent ISCHEMIA trial, which did not show benefit of initial invasive strategy compared with initial conservative strategy among patients with stable coronary disease and moderate or severe ischemia^17^, selecting more appropriate candidates for PCI has become an important and challenging question in daily practice. To date, well-validated FFR-guided strategy remains as a standard approach to manage patients with intermediate coronary stenosis and current guidelines recommend revascularization for patients with FFR ≤ 0.80^1,2^. However, it should be noted that FFR is a pressure-derived surrogate of coronary flow and the current results suggest that CFR can be used to assist and potentially improve the FFR-guided strategy by providing further information to select patients who are able to be medically managed despite abnormal FFR value. These results re-emphasize the importance of comprehensive physiologic assessment with combined measurement of coronary pressure and flow, and an individualized approach for decision of revascularization. However, the current study should be regarded as a hypothesis-generating study due to the limitations as described below.

Some limitations of the current study should be noted. First, since the decision to perform or defer PCI was at the discretion of the operator, there is the possibility of selection bias. Second, the inherent limitations of nonrandomized comparisons, such as allocation bias and uneven distribution of risk factors, should be considered. In addition, there were significant differences in lesion length, diameter stenosis, and FFR in the target vessels. Although multiple sensitivity analyses were performed to adjust for baseline differences and showed similar results, unmeasured variables and other potential confounders could not be completely controlled. Third, most patients in the deferred group had gray zone FFR values (0.75–0.80) and thus generalization of the results to the patients with extremely low FFR and preserved CFR would be limited. Fourth, it is well known that one of the most important treatment effects of PCI is relief of symptoms. Since the current registry did not systematically collect the data on patients’ symptoms after PCI or deferral, this could not be evaluated. Further study is required to clarify this issue. Fifth, there was no detailed information on the medication profiles of enrolled patients during the follow-up period. Sixth, the number of cases and events, especially in the deferred group, was not enough for comprehensive statistical analysis, although the study population was derived from the large international registry.

In conclusion, the current hypothesis-generating study showed that there was no significant difference in clinical outcomes following an operator-based decision on PCI between the revascularization and deferred groups among patients with intermediate stenosis with FFR ≤ 0.80 but CFR > 2.0. However, there was statistically non-significant trend of favoring PCI in patients with FFR < 0.75. Further well-designed study is warranted to confirm this finding.

## Supplementary information

Supplementary information

## Abbreviations

CFR: Coronary flow reserve
CI: Confidence intervals
FFR: Fractional flow reserve
HR: Hazard ratio
MACE: Major adverse cardiac events
MI: Myocardial infarction
PCI: Percutaneous coronary intervention
