# Supplementary material for: Non-randomized comparison between revascularization and deferral for intermediate coronary stenosis with abnormal fractional flow reserve and preserved coronary flow reserve
Source: Sci Rep. 2021 Apr 28;11:9126. doi: 10.1038/s41598-021-88732-4 (PMC8080642; doi:10.1038/s41598-021-88732-4)

**Supplementary Appendix**

**Non-Randomized Comparison between Revascularization and Deferral for Intermediate Coronary Stenosis with Abnormal Fractional Flow Reserve and Preserved Coronary Flow Reserve**

Doosup Shin, MD^1^, Joo Myung Lee, MD, MPH, PhD^2, *^, Seung Hun Lee, MD, PhD^2^, Doyeon Hwang, MD^3^, Ki Hong Choi, MD^2^, Hyun Kuk Kim, MD, PhD^4^, Joon-Hyung Doh, MD, PhD^5^, Chang-Wook Nam, MD, PhD^6^, Eun-Seok Shin, MD, PhD^7^, Masahiro Hoshino, MD^8^, Tadashi Murai, MD, PhD^8^, Taishi Yonetsu, MD^9^, Hernán Mejía-Rentería, MD^10^, Tsunekazu Kakuta, MD, PHD^8^, Javier Escaned, MD, PhD^10,11^, Bon-Kwon Koo, MD, PhD^3,12, *^

^1^Division of Cardiovascular Medicine, Department of Internal Medicine, University of Iowa Carver College of Medicine, Iowa City, IA, USA; ^2^Division of Cardiology, Department of Internal Medicine, Heart Vascular Stroke Institute, Samsung Medical Center, Sungkyunkwan University School of Medicine, Seoul, Korea; ^3^Department of Internal Medicine and Cardiovascular Center, Seoul National University Hospital, Seoul, Korea; ^4^Department of Internal Medicine and Cardiovascular Center, Chosun University Hospital, University of Chosun College of Medicine, Gwangju, Korea; ^5^Department of Medicine, Inje University Ilsan Paik Hospital, Goyang, Korea; ^6^Department of Medicine, Keimyung University Dongsan Medical Center, Daegu, Korea; ^7^Division of Cardiology, Ulsan Medical Center, Ulsan, Korea; ^8^Division of Cardiovascular Medicine, Tsuchiura Kyodo General Hospital, Ibaraki, Japan; ^9^Department of Cardiovascular Medicine, Tokyo Medical and Dental University, Tokyo, Japan; ^10^Cardiovascular Institute, Hospital Clinico San Carlos, Madrid, Spain; ^11^Centro Nacional de Investigaciónes Cardiovasculares Carlos III (CNIC), Madrid, Spain; ^12^Institute on Aging, Seoul National University, Seoul, Korea

* Corresponding authors

**Contents**

**- Supplementary Tables**

**- Supplementary Figures and Figure Legends**

**Supplementary Tables**

**Supplementary Table 1. Baseline Characteristics of Patients and Lesions in Deferred Group According to Clinical Events**

|  | **Total** | **No Event** | **Event** | **P value** |
| --- | --- | --- | --- | --- |
| **Per-patient analysis (n=99)** | **99** | **88** | **11** |  |
| **Demographics** |  |  |  |  |
| Age | 64.5 (57.0-71.0) | 61.5 (56.5-68.0) | 67.0 (61.0-72.0) | 0.175 |
| Female | 12 (12.1) | 10 (11.4) | 2 (18.2) | 0.619 |
| **Cardiovascular risk factors** |  |  |  |  |
| Hypertension | 69 (69.7) | 65 (73.9) | 4 (36.4) | 0.017 |
| Diabetes mellitus | 28 (28.3) | 24 (27.3) | 4 (36.4) | 0.500 |
| Hyperlipidemia | 68 (68.7) | 61 (69.3) | 7 (63.6) | 0.736 |
| Current smoker | 23 (23.2) | 21 (23.9) | 2 (18.2) | 1.000 |
| Family history of CAD | 9 (9.5) | 8 (9.4) | 1 (10.0) | 1.000 |
| **Clinical presentation** |  |  |  | **0.365** |
| Acute coronary syndrome | 15 (15.2) | 12 (13.6) | 3 (27.3) |  |
| Stable ischemic heart disease | 84 (84.9) | 76 (86.4) | 8 (72.7) |  |
| **Per-vessel analysis (n=105)** | **105** | **95** | **10** |  |
| **Target vessel location** |  |  |  | **0.543** |
| Left anterior descending artery | 92 (88.5) | 85 (89.5) | 7 (77.8) |  |
| Left circumflex artery | 7 (6.7) | 6 (6.3) | 1 (11.1) |  |
| Right coronary artery | 5 (4.8) | 4 (4.2) | 1 (11.1) |  |
| **Quantitative coronary angiography** |  |  |  |  |
| Reference vessel diameter, mm | 2.88 (2.38-3.19) | 2.88 (2.38-3.15) | 2.84 (2.41-4.12) | 0.344 |
| Minimal luminal diameter, mm | 1.48 (1.17-1.71) | 1.48 (1.18-1.71) | 1.60 (1.10-1.78) | 0.873 |
| Diameter stenosis, % | 47.7 ± 12.8 | 47.2 ± 12.9 | 52.0 ± 11.6 | 0.266 |
| Lesion length, mm | 10.00 (6.16-18.06) | 9.86 (6.09-18.16) | 12.96 (9.17-16.50) | 0.430 |
| **Invasive physiologic indices** |  |  |  |  |
| FFR, Pre-PCI | 0.78 (0.75-0.79) | 0.78 (0.75-0.79) | 0.77 (0.73-0.79) | 0.624 |
| CFR, Pre-PCI | 3.14 (2.58-3.90) | 3.10 (2.55-3.90) | 3.65 (3.00-4.47) | 0.292 |

Values are mean ± standard deviation, median (interquartile range), or number (%).

Abbreviations: CAD, coronary artery disease, CFR, coronary flow reserve; FFR, fractional flow reserve; PCI, percutaneous coronary intervention.

**Supplementary Table 2. Comparison of Clinical Outcome According to Treatment Strategy**

| **Per-vessel analysis** | **PCI**  **(N = 233)** | **Deferred**  **(N = 105)** | **Unadjusted HR**  **(95% CI)** | **P value** | **Adjusted HR^*^**  **(95% CI)** | **P value** |
| --- | --- | --- | --- | --- | --- | --- |
| **Vessel-specific MI** | 1.7 (2) | 1.2 (1) | 0.928 (0.069-12.41) | 0.955 | 0.639 (0.033-12.526) | 0.768 |
| **Vessel-specific revascularization** | 6.9 (12) | 8.0 (6) | 0.998 (0.382-2.608) | 0.997 | 1.044 (0.355-3.073) | 0.938 |
| **Vessel-specific MI or revascularization** | 8.1 (13) | 8.0 (6) | 0.899 (0.344-2.349) | 0.829 | 0.932 (0.321-2.712) | 0.898 |

Data expressed as cumulative incidence of clinical outcomes and number of events. Cumulative incidence of clinical outcomes represents Kaplan-Meier estimates during median follow-up of 1286.0 days (Q1-Q3 733.0-1693.0 days). P values for log-rank test in survival analysis.

^*^Adjusted for age, sex, diabetes mellitus, hypertension, hypercholesterolemia, current smoking, and acute coronary syndrome.

Abbreviations: CI, confidence interval; HR, hazard ratio; MI, myocardial infarction; PCI, percutaneous coronary intervention.

**Supplementary Table 3. Sensitivity Analysis regarding Clinical Outcome According to Treatment Strategy**

| **Per-vessel analysis** | **PS^*^-adjusted HR**  **(95% CI)** | **P value** | **IPW adjusted HR**  **(95% CI)** | **P value** | **PS^*^-matched HR**  **(95% CI)** | **P value** |
| --- | --- | --- | --- | --- | --- | --- |
| **Vessel-specific MI** | 0.381 (0.023 – 6.389) | 0.503 | 1.185 (0.052 – 26.882) | 0.915 | 0.522 (0.047– 3.997) | 0.526 |
| **Vessel-specific revascularization** | 1.763 (0.421 – 7.378) | 0.438 | 0.913 (0.319 – 2.614) | 0.865 | 1.390 (0.406-4.758) | 0.600 |
| **Vessel-specific MI or revascularization** | 1.283 (0.294-5.590) | 0.740 | 0.805 (0.281-2.305) | 0.686 | 1.073 (0.329-3.499) | 0.908 |

^*^Propensity score was calculated based on multiple logistic regression model after adjusting for age, sex, hypertension, diabetes, hypercholesterolemia, smoking, clinical presentation, vessel location, percent diameter stenosis, lesion length, pre-intervention fractional flow reserve, and pre-intervention coronary flow reserve.

Abbreviations: CI, confidence interval; HR, hazard ratio; IPW, inverse probability weight; MI, myocardial infarction; PS, propensity score.

**Supplementary Table 4. Independent Predictors of Major Adverse Cardiac Events During 5 Years of Follow Up^†^**

| **Variable** | **Univariate analysis** | | **Multivariable analysis^*^** | |
| --- | --- | --- | --- | --- |
|  | **HR (95% CI)** | **P value** | **HR (95% CI)** | **P value** |
| **Target vessel intervention** | **0.865 (0.413-1.809)** | **0.700** | **0.463 (0.187-1.144)** | **0.095** |
| **Age** | 1.007 (0.969-1.048) | 0.709 | 1.021 (0.977-1.067) | 0.356 |
| **Male** | 0.605 (0.232-1.575) | 0.303 | 0.537 (0.200-1.446) | 0.219 |
| **Hypertension** | 0.552 (0.272-1.119) | 0.100 | 0.528 (0.247-1.125) | 0.098 |
| **Hypercholesterolemia** | 0.887 (0.425-1.853) | 0.750 | 0.905 (0.421-1.945) | 0.798 |
| **Diabetes mellitus** | 1.606 (0.787-3.279) | 0.193 | 2.043 (0.965-4.323) | 0.062 |
| **Current smoking** | 1.681 (0.791-3.573) | 0.177 | 1.553 (0.693-3.481) | 0.285 |
| **Percent diameter stenosis, pre-PCI** | 1.012 (0.984-1.040) | 0.413 | 1.014 (0.983-1.046) | 0.369 |
| **Lesion length, pre-PCI** | 1.019 (0.984-1.055) | 0.294 | 1.018 (0.981-1.057) | 0.349 |
| **FFR, pre-PCI, per 0.01 increase** | 0.969 (0.919-1.022) | 0.242 | 0.950 (0.888-1.016) | 0.133 |
| **CFR, pre-PCI** | 1.156 (0.818-1.635) | 0.412 | 1.248 (0.881-1.767) | 0.213 |

^*^Calculated using multivariable Cox proportional hazard model. C-index of the model was 0.690 (95% confidence interval: 0.577-0.803)

^†^ MACE included all-cause death, target-vessel MI, and target-vessel revascularization.

Abbreviations: CFR, coronary flow reserve; CI, confidence interval; FFR, fractional flow reserve; HR, hazard ratio; PCI, percutaneous coronary intervention.

**Supplementary Table 5. Independent Predictors of Vessel-Specific MI or Revascularization During 5 Years of Follow Up**

| **Variable** | **Univariate analysis** | | **Multivariable analysis^*^** | |
| --- | --- | --- | --- | --- |
|  | **HR (95% CI)** | **P value** | **HR (95% CI)** | **P value** |
| **Target vessel intervention** | **1.112 (0.426-2.904)** | **0.829** | **0.807 (0.161-4.045)** | **0.795** |
| **Age** | 0.963 (0.930-0.998) | 0.038 | 0.972 (0.924-1.024) | 0.285 |
| **Male** | 0.598 (0.174-2.056) | 0.415 | 0.461 (0.125-1.705) | 0.246 |
| **Hypertension** | 0.602 (0.241-1.505) | 0.278 | 0.589 (0.194-1.789) | 0.350 |
| **Hypercholesterolemia** | 1.075 (0.406-2.848) | 0.885 | 1.098 (0.368-3.274) | 0.867 |
| **Diabetes mellitus** | 1.289 (0.508-3.269) | 0.593 | 1.639 (0.640-4.199) | 0.303 |
| **Current smoking** | 2.028 (0.800-5.143) | 0.136 | 1.515 (0.533-4.301) | 0.436 |
| **Percent diameter stenosis, pre-PCI** | 1.010 (0.979-1.041) | 0.544 | 1.009 (0.975-1.045) | 0.601 |
| **Lesion length, pre-PCI** | 1.023 (0.972-1.076) | 0.390 | 1.019 (0.963-1.077) | 0.521 |
| **FFR, pre-PCI, per 0.01 increase** | 0.969 (0.907-1.036) | 0.354 | 0.971 (0.874-1.079) | 0.590 |
| **CFR, pre-PCI** | 1.313 (0.881-1.958) | 0.181 | 1.245 (0.890-1.740) | 0.200 |

^*^Calculated using multivariable Cox proportional hazard model. C-index of the model was 0.710 (95% confidence interval: 0.595-0.826)

Abbreviations: CFR, coronary flow reserve; CI, confidence interval; FFR, fractional flow reserve; HR, hazard ratio; MI, myocardial infarction; PCI, percutaneous coronary intervention.

**Supplementary Figure Legends**

**Supplementary Figure 1. Distribution of FFR, CFR, and Diameter Stenosis**

Distributions of (A) Fractional flow reserve, (B) Coronary flow reserve, (C) Diameter stenosis are presented.

Abbreviations: CFR, coronary flow reserve; FFR, fractional flow reserve.

**Supplementary Figure 2. Comparison of Vessel-Specific Clinical Events According to Treatment Strategy**

Kaplan-Meier curves and cumulative incidence of (A) vessel-specific myocardial infarction and (B) vessel-specific revascularization were compared according to treatment strategy (PCI or deferral of revascularization). Adjusted HR and 95% CI were calculated based on multivariable Cox proportional hazard regression model. Adjusted variables included age, sex, diabetes mellitus, hypertension, hyperlipidemia, current smoking, and presentation with acute coronary syndrome.

Abbreviations: CI, confidence interval; HR_adj_, adjusted hazard ratio; PCI, percutaneous coronary intervention.

**Supplementary Figure 3. Subgroup Analysis for Vessel-Specific MI or Revascularization**

Comparison of vessel-specific MI or revascularization according to treatment strategy was performed within various subgroups. Adjusted HR and 95% CI were calculated based on multivariable Cox proportional hazard regression model. Adjusted variables included age, sex, diabetes, hyperlipidemia, current smoking, and presentation with acute coronary syndrome, as appropriate.

Abbreviations: CFR, coronary flow reserve; CI, confidence interval; DM, diabetes mellitus; FFR, fractional flow reserve; HLD, hyperlipidemia; HR, hazard ratio; MI, myocardial infarction; PCI, percutaneous coronary intervention.


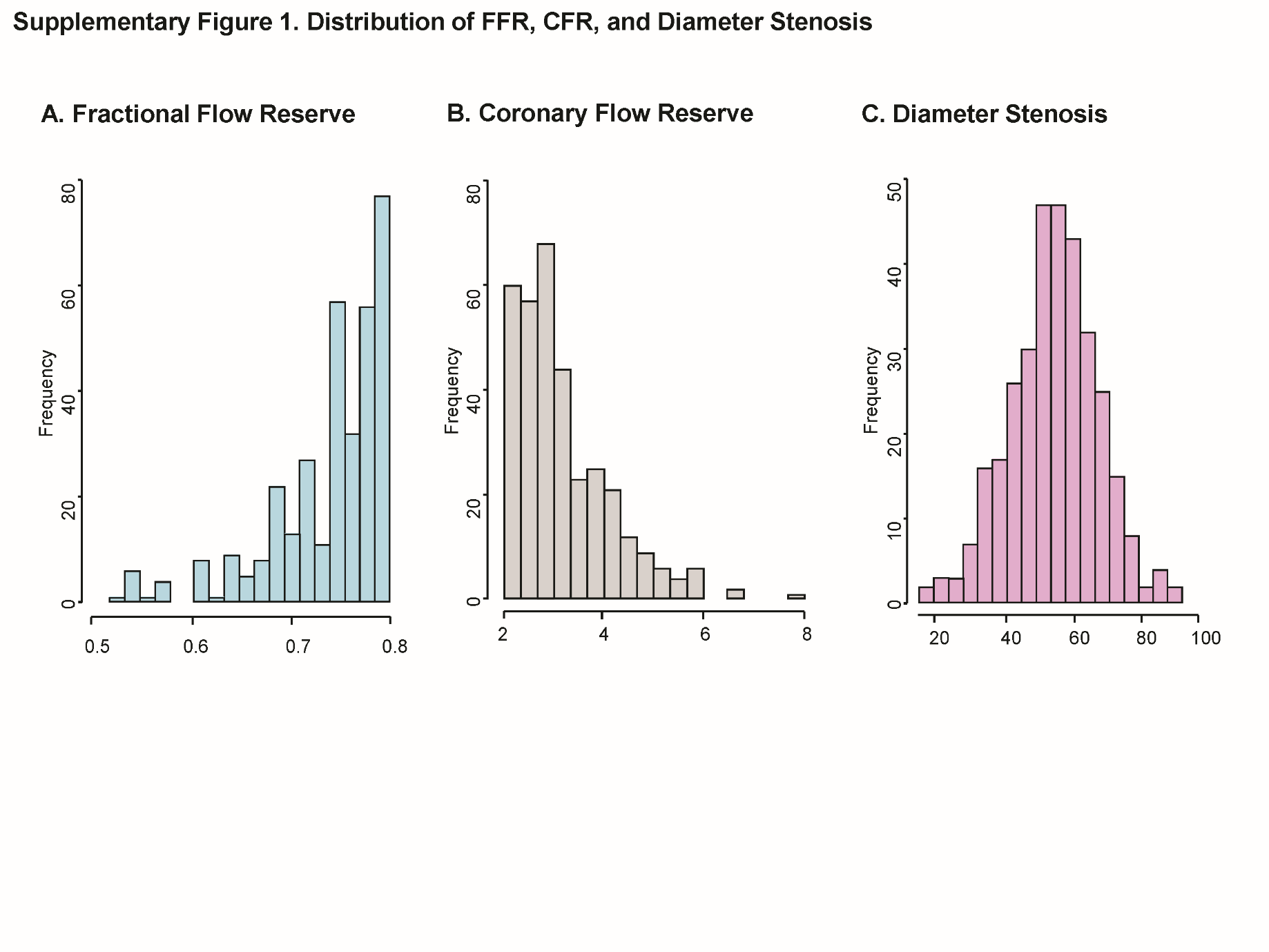

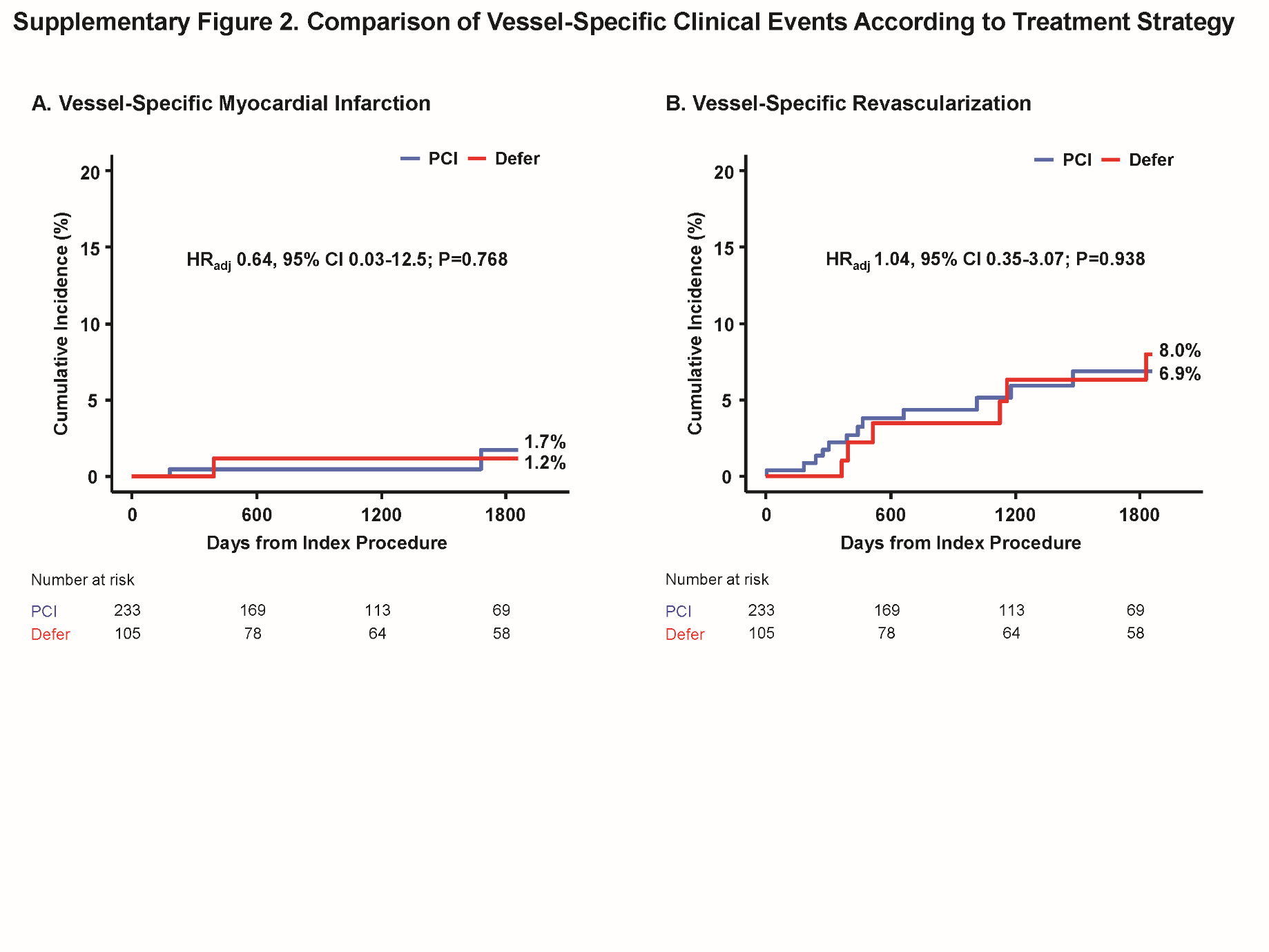


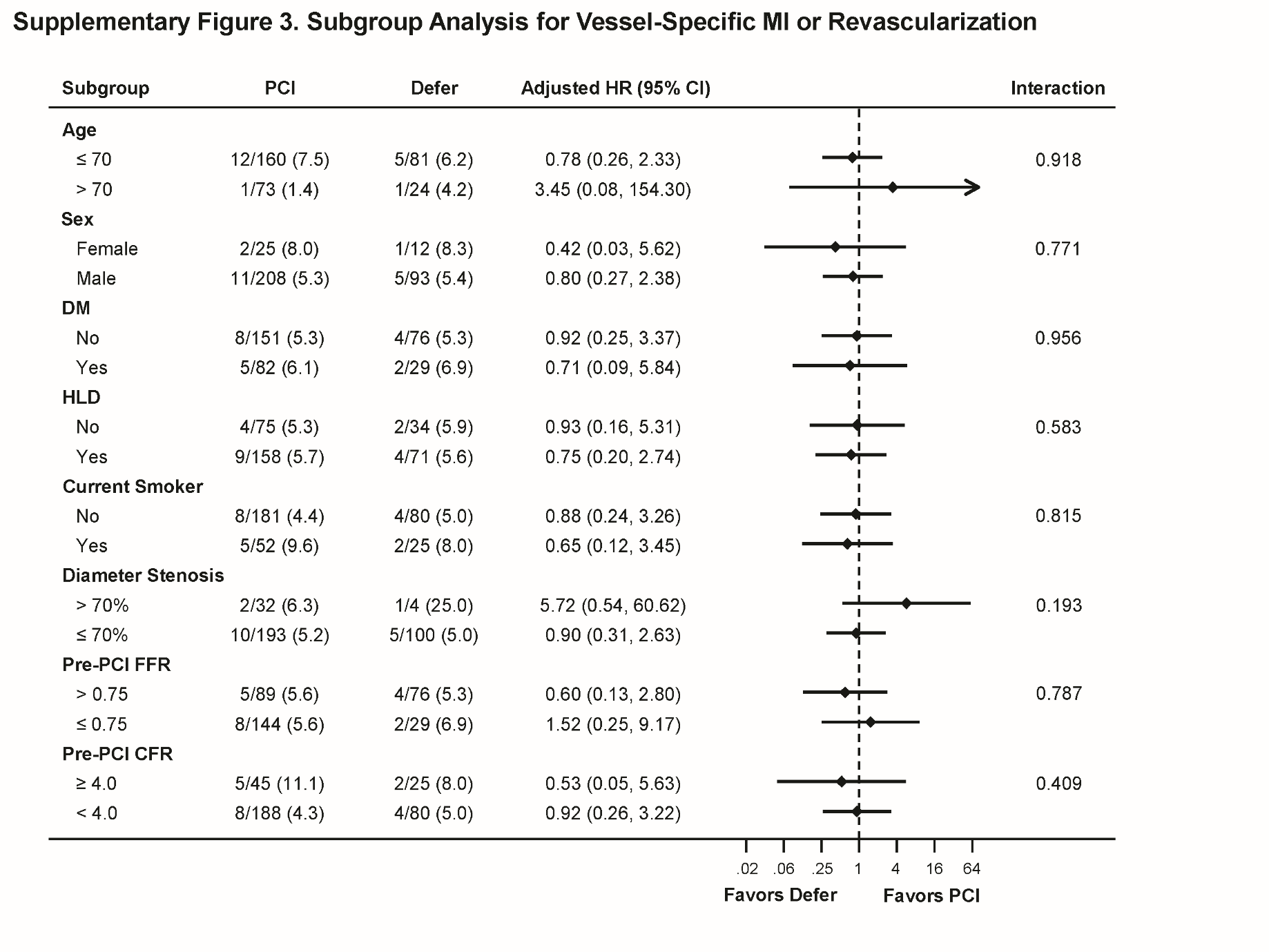

Supplement: Supplementary file 1 — Supplementary information [file 41598_2021_88732_MOESM1_ESM.docx]
